# Structural Characteristics, Antioxidant, and Immunostimulatory Activities of an Acidic Polysaccharide from Raspberry Pulp

**DOI:** 10.3390/molecules27144385

**Published:** 2022-07-08

**Authors:** Yongjing Yang, Xingxing Yin, Dejun Zhang, Benyin Zhang, Jie Lu, Xuehong Wang

**Affiliations:** 1 College of Ecological and Environmental Engineering, Qinghai University, Xining 810016, China; y200713000515@qhu.edu.cn (X.Y.); 1998990035@qhu.edu.cn (D.Z.); benyinzhang@qhu.edu.cn (B.Z.); ys210854000307@qhu.edu.cn (J.L.); ys210713000141@qhu.edu.cn (X.W.); 2 State Key Laboratory of Plateau Ecology and Agriculture, Qinghai University, Xining 810016, China

**Keywords:** raspberry pulp, acidic polysaccharide, isolation, structural characterization, antioxidant activity, immunostimulatory activity

## Abstract

The extraction and characterization of new bioactive plant-derived polysaccharides with the potential for use as functional foods and medicine have attracted much attention. In the present study, A novel acidic polysaccharide (RPP-3a) with a weight-average molecular weight (Mw) of 88,997 Da was isolated from the raspberry pulp. RPP-3a was composed of rhamnose, arabinose, galactose, glucose, mannose, and galacturonic acid at a molar ratio of 13.1:28.6:16.8:1.4:6.2:33.9. Structural analysis suggested that the RPP-3a backbone was composed of repeating units of →4)-*β*-Gal*p*-(1→3,4)-*α*-Rha*p*-(1→[4)-*α*-GalA*p*-(1→4)-*α*-GalA*p*-(1→]_n_ with branches at the C-4 position of rhamnose. The side chain of RPP-3a, containing two branch levels, was comprised of *α*-Ara*f*-(1→, →5)-*α*-Ara*f*-(1→, →3,5)-*α*-Ara*f*-(1→, →3)-*β*-Gal*p*-(1→, →3,6)-*β*-Gal*p*-(1→, →4)-*β*-Glc*p*-(1→, and →2,6)-*α*-Man*p*-1→ residues. RPP-3a exhibited moderate reducing power and strong hydroxyl and superoxide anion radical scavenging abilities. RPP-3a significantly promoted the viability of RAW264.7 macrophages by increasing the production of nitric oxide (NO), tumor necrosis factor-α (TNF-α), interleukin-6 (IL-6), and interleukin-1β (IL-1β) at both the expression and transcriptional levels. In summary, the immunostimulatory and antioxidant activities make RPP-3a a viable candidate as a health-beneficial functional dietary supplement.

## 1. Introduction

As a member of the berry family, raspberry (*Rubus idaeus* L.) is widely distributed and cultivated worldwide [1]. Its fruit, sub-globose and bright red, is also known as “fu-pen-zi” in China and is commonly used in traditional Chinese medicine to treat urination frequency, kidney enuresis, and other illnesses [2]. Presently, raspberry is considered a green fruit and has gained more acceptance due to its high nutritional value. Raspberry fruit contains numerous bioactive compounds, including flavonoids, tannins, phenolic acids, stilbenoids, polysaccharides, vitamins, and minerals [3], and dietary intake of them has the advantage of treating cardiovascular diseases, obesity, cancer, and degenerative diseases [4].

Polysaccharides, a class of carbohydrates, consist of numerous monosaccharides joined by glycosidic bonds in branched or unbranched chains. They are usually considered the primary and active components in plants, animals, and microorganisms [5]. Accumulating evidence suggests that polysaccharides extracted from plants, with relatively low toxicity and side effects, exert diverse biological activity, such as antitumor, anti-inflammatory, antioxidant, antimicrobial, antidiabetic effect, and immunomodulatory effects [6,7,8]. Hence, the extraction and characterization of new bioactive plant-derived polysaccharides with the potential for use as functional foods and medicine have attracted much attention. Coincidentally, polysaccharides are one of the most important bioactive components in raspberries. In recent years, the biological activities of polysaccharides derived from raspberry fruit have been reported in several studies. The crude polysaccharides from raspberry fruit possessed antioxidant, anti-inflammatory, anticancer activity, and even detoxification effect [9,10]. Our previous study found that crude raspberry pulp polysaccharides (RPPs) exhibited significant antitumor activity and chemotherapy enhancement in vivo by increasing the cellular immune response of the host organism without any lesions in the liver or kidney tissues [11]. Along with continuous research, more and more homogeneous bioactive polysaccharides have been separated from raspberries. Ke et al. (2019) reported that a heteropolysaccharide separated from raspberry fruit afforded protection against palmitic acid-induced lipotoxicity in human hepatocytes [3]. A heteropolysaccharide (RCP I) and a degraded polysaccharide (DRCP I) were obtained from raspberry fruits, both of which displayed high antioxidant activity [12]. In our recent study, a novel acidic polysaccharide named RPP-2a was isolated from raspberry pulp and exhibited significant macrophage activation activity [13]. These results indicated that the polysaccharides derived from raspberries have great potential for in-depth development and utilization. In the present work, another acidic polysaccharide was isolated from raspberry pulp, and its structural information was systematically characterized. Its antioxidant and immunoregulatory activities were also investigated.

## 2. Results

### 2.1. Isolation and Purification of Homogeneous Acidic Polysaccharide RPP-3a

**Figure 1:**
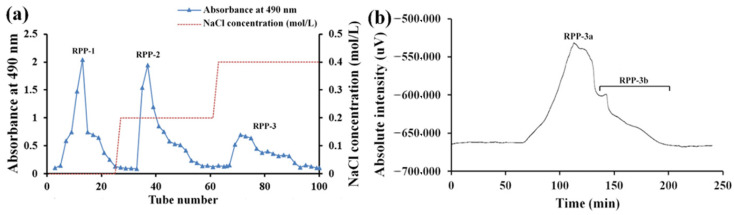
Elution profiles of RPPs on DEAE-Sepharose fast flow chromatography (**a**) and RPP-3 on Sephadex G-200 column (**b**).

Crude polysaccharide RPPs were separated by DEAE-Sepharose fast flow chromatography according to different polarities of polysaccharides. As depicted in Figure 1a, three fractions were collected from DEAE-Sepharose fast flow chromatography, named RPP-1, RPP-2, and RPP-3, which were eluted with deionized water, 0.2 M, and 0.4 M NaCl solutions, respectively. RPP-3 was further purified by a Sephadex G-200 column according to the molecular weight (Figure 1b), and two peaks (RPP-3a and RPP-3b) were observed. However, further purification work was needed due to the heterogeneity of RPP-3b. Thus, RPP-3a was concentrated, dialyzed, and lyophilized for further analysis.

### 2.2. Homogeneity and Molecular Weight of RPP-3a

**Figure 2:**
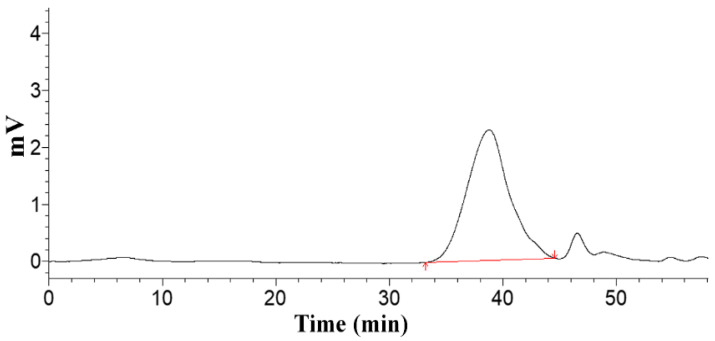
The HPGPC profile of RPP-3a.

A single symmetrical peak was obtained from high-performance gel permeation chromatography (HPGPC), suggesting the molecular weight homogeneity and the purity of RPP-3a (Figure 2). The weight-average molecular weight (Mw), number-average molecular weight (Mn), and peak molecular weight (Mp) of RPP-3a were 88,997 Da, 59,284 Da, and 70,366 Da, respectively. Mw/Mn is a measure of the width of the molecular weight distribution [14], and the Mw/Mn value of RPP-3a was 1.501.

### 2.3. Fourier Transform Infrared Spectrophotometer Spectrum (FT-IR) of RPP-3a

**Figure 3:**
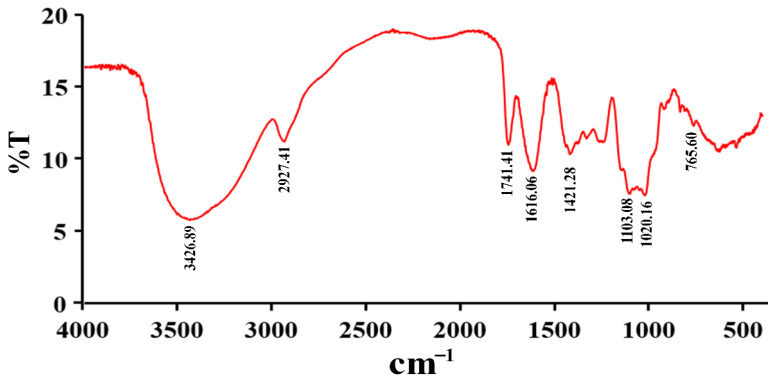
FT-IR spectra of RPP-3a in a range from 400 to 4000 cm^−1^.

The FT-IR spectrum of RPP-3a is illustrated in Figure 3. RPP-3a displayed the typical absorption peaks of polysaccharides at 3426.89 cm^−1^, 2927.41 cm^−1^, and 1421.28 cm^−1^ [15]. The band in the region of 3426.89 cm^−1^ indicated the characteristic absorption of -OH stretching vibration [16,17]. The absorption peaks near 2927.41 cm^−1^ and 1421.28 cm^−1^ were caused by the stretching vibration of C-H [18]. The peaks at 1741.41 and 1616.06 cm^−1^ corresponded to symmetric and asymmetric C=O stretching vibrations [19]. The absorption band at 765.60 cm^−1^ suggested the presence of a pyranose structure [20]. The peaks at 1103.08 cm^−1^ and 1020.18 cm^−1^ were assigned to C-O and O-H stretching vibrations, respectively [21].

### 2.4. The Monosaccharide Composition of RPP-3a

**Figure 4:**
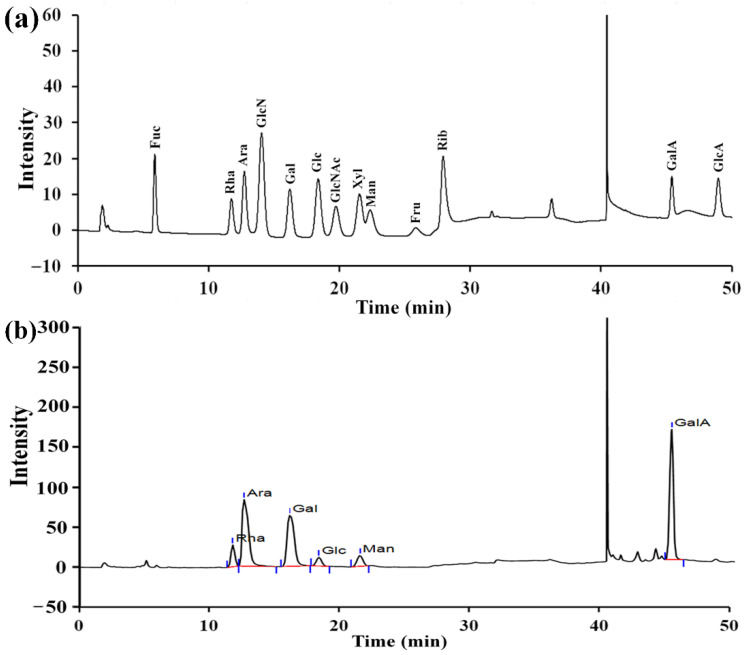
Monosaccharide composition of RPP-3a. The anion-exchange chromatography profile of mixed monosaccharide standards (**a**) and monosaccharides in RPP-3a (**b**).

As depicted in Figure 4a, RPP-3a was composed of rhamnose (Rha), arabinose (Ara), galactose (Gal), glucose (Glc), mannose (Man), and galacturonic acid (GalA) at a molar ratio of 13.1:28.6:16.8:1.4:6.2:33.9. The content of GalA was the highest among the monosaccharides.

### 2.5. Methylation and Gas Chromatography-Mass Spectrometry (GC-MS) Analysis of RPP-3a

**Figure 5:**
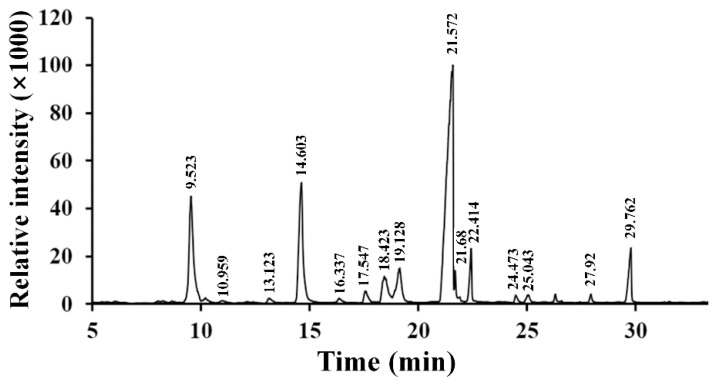
The GC-MS chromatogram of PMAAs of RPP-3a.

Methylation analysis, a classical method, was used to investigate the glycosidic linkage types of the monosaccharides. As depicted in Figure 5, a total of fifteen methylated monosaccharides were identified in the GC-MS chromatogram, suggesting that RPP-3a was composed of fifteen glycosyl linkages patterns. Information on methylated sugar, mass fragment information, glycosyl linkage patterns, and molar ratios is summarized in Table 1. Among these connection modes, the molar ratios of →5)-Ara*f*-(1→, →3,5)-Ara*f*-(1→, →2,6)-Man*p*-(1→, →4)-Gal*p*-(1→, →3)-Gal*p*-(1→, and →3,6)-Gal*p*-(1→ were 15.62, 4.48, 5.10, 45.41, 3.47, and 4.72, respectively. The non-reducing terminals were mainly composed of Ara*f*-(1→ with a molar ratio of 14.73. The above results indicated that RPP-3a was a branched heteropolysaccharide.

### 2.6. Nuclear Magnetic Resonance (NMR) Spectra of RPP-3a

**Figure 6:**
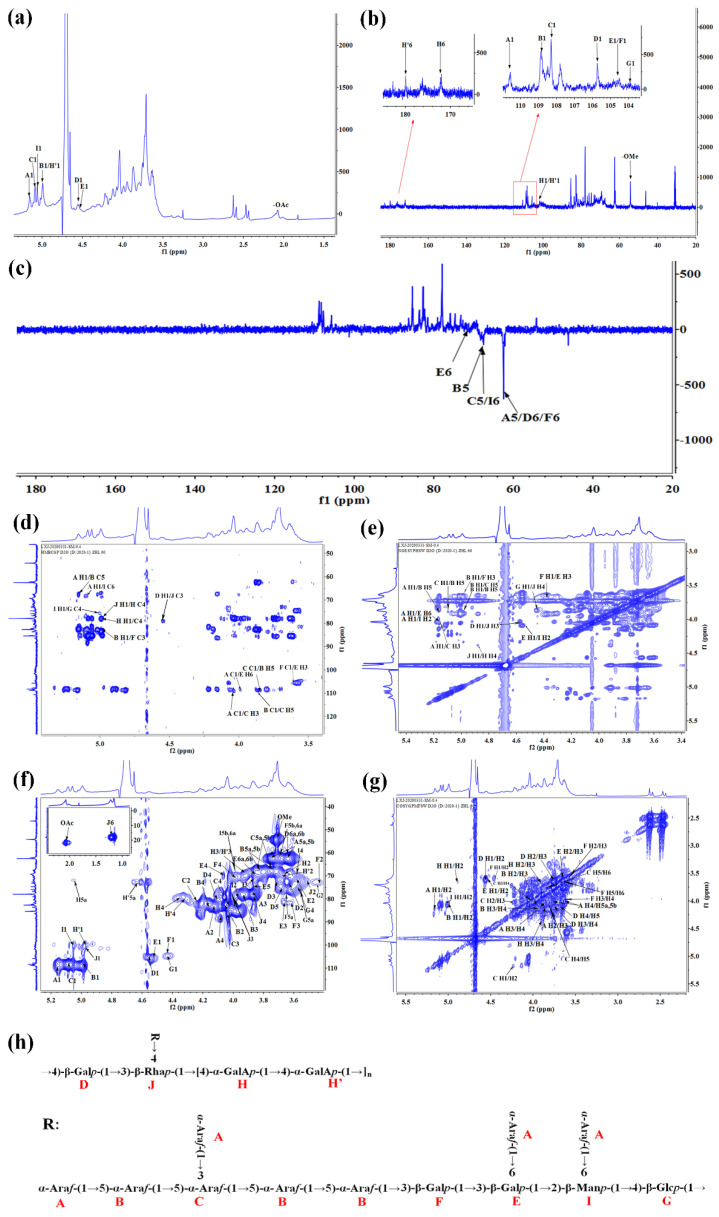
NMR spectra and proposed structure of RPP-3a. ^1^H NMR (**a**), ^13^C NMR (**b**), DEPT-135 (**c**), HMBC (**d**), NOESY (**e**), HSQC (**f**), COSY (**g**), and the structure of RPP-3a (**h**).

^1^H-NMR and ^13^C-NMR signals were assigned according to correlations in the 2D-NMR spectra to interpret the structure of RPP-3a. As shown in Figure 6a, proton spectrum signals were mainly concentrated between 3.0 and 5.5 ppm, and the signals of the anomeric proton were distributed in the δ_H_ 4.4–5.2 ppm region. The nuclear magnetic carbon signals were mainly concentrated from 60 to 120 ppm, and the main anomeric carbon signal peaks were mainly in δ_C_ 100–110 ppm (Figure 6b). In general, the anomeric proton signal between δ_H_ 4.4 and 5.0 ppm or anomeric carbon signal between δ_C_ 103 and 105 ppm suggested the presence of *β*-configuration pyranose units, and the anomeric proton signal over δ_H_ 4.9 ppm or anomeric carbon signal between δ_C_ 99 and 101 ppm suggested the presence of *α*-configuration pyranose units [22,23]. In addition, the anomeric carbon signal of the *α*-configuration furanose unit is usually between δ_C_ 107 and 110 ppm [24,25]. Although not all of the NMR signals could not be identified due to the complexity of the spectrum, several characteristic peaks of the Ara, Gal, Glc, GalA, Man, and Rha residues were assigned. The results are listed in Table 2. A total of 11 glycosyl residues were found in RPP-3a and designated as residues A, B, C, D, E, F, G, H, H’, I, and J for convenience. Residues A, B, and C were *α*-configuration furanose units and were identified as *α*-Ara*f*-(1→, →5)-*α*-Ara*f*-(1→ and →3,5)-*α*-Ara*f*-(1→ by the anomeric signals at δ_H_/δ_C_ 5.16/110.45, 4.98/108.83, and 5.1/108.28, respectively. Residues D, E, F, and G all belonged to *β*-configuration pyranose. →4)-*β*-Gal*p*-(1→ (D), →3,6)-*β*-Gal*p*-(1→ (E), and →3)-*β*-Gal*p*-(1→ (F) were matched to the signals of δ_H_/δ_C_ 4.56/105.74, 4.52/104.69 and 4.4/104.48, respectively. Similar signals of anomeric proton and carbon have been reported in some literature [26,27]. Residue G was found to have a downfield H-1 (4.45 ppm) and a downfield C-1 (103.63 ppm), consistent with →4)-*β*-Glc*p*-(1→ [28]. A carbon signal between δ_C_ 160 and 180 ppm suggested the presence of a carboxyl group of uronic acid [29]. According to the anomeric signals at δ_H_/δ_C_ 4.84/100.81 and 4.99/100.39, as well as the carbon signals at δ_C_ 172.19 and 176.66 ppm, residues H and H’ were assigned to the methyl-esterified →4)-*α*-GalA*p*-(1→ and →4)-*α*-GalA*p*-(1→, respectively. Additionally, the signal at δ_H_ 3.74/δ_C_ 54.4 ppm was assigned to the oxygen methyl of *α*-Gal*p*A in the C-2 or C-3 position [30]. Residue I and J were *α*-configuration pyranose units and were identified as →2,6)-*α*-Man*p*-1→ and →3,4)-*α*-Rha*p*-(1→ by the anomeric signals at δ_H_/δ_C_ 5.05/99.62 and δ_H_/δ_C_ 4.9/99.5, respectively [31,32]. In the DEPT-135 spectra, the signals at δ 62.64, 68.09, and 67.82 ppm were assigned to the C-5 of A, B, and C, respectively, and the signals at δ 62.11, 70.76, 62.26, and 67.2 ppm were assigned to the C-6 of D, E, F, and I, respectively.

The HMBC and NOESY spectrum were further analyzed to determine the sugar sequences and linkage positions of RPP-3a (Figure 6d,e). The cross-peaks between the H1 (δ 4.56) of D and the C3 (δ 77.46) of J in the HMBC spectrum and H1 (δ 4.56) of D and the H3 (δ 3.97) of J in NOESY spectrum revealed the connection of →4)-*β*-Gal*p*-(1→3,4)-*α*-Rha*p*-(1→. The correlation peak in the HMBC map between the C4 (δ 80.06) of H and the H1 (δ 4.9) of J and the cross-peak in the NOESY spectrum between the H1 (δ 4.9) of J and the H4 (δ 4.36) of H suggesting the presence of the glycosidic bond →3,4)-*α*-Rha*p*-(1→4)-*α*-GalA*p*-(1→. The cross-peak between the H1 (δ 4.84) of H and the H4 (δ 4.33) of H’ in the NOESY spectrum indicated the presence of a →4)-*α*-GalA*p*-(1→4)-*α*-GalA*p*-(1→ fragment. The C5 (δ 68.09) of B was associated with the H1 (δ 5.16) of A in the HMBC spectrum, and the H5 (δ 3.84) of B was associated with the H1 (δ 5.16) of A in the NOESY spectrum suggested the presence of an *α*-Ara*p*-(1→5)-*α*-Ara*p*-(1→ linkage. The correlation between the C1 (δ 108.83) of B and the H5 (δ 3.84) of C (HMBC map), and the H1 (δ 4.98) of B and the H5 (δ 3.84) of C (NOESY map) suggested the presence of →5)-*α*-Ara*p*-(1→3,5)-*α*-Ara*f*-(1→. The C1 (δ 108.28) of C and the H5 (δ 3.84) of B (HMBC map), as well as the H1 (δ 5.1) of C and the H5 (δ 3.84) of B (NOESY map), indicated the presence of →3,5)-*α*-Ara*f*-(1→5)-*α*-Ara*p*-(1→ linkages. The correlation peaks between the C1 (δ 110.45) of A and the H3 (δ 4.05) of C in the HMBC spectrum, and the H1 (δ 5.16) of A and the H3 (δ 4.05) of C in NOESY spectrum suggested the presence of the glycosidic bond *α*-Ara*f*-(1→3,5)-*α*-Ara*f*-(1→. The manner of the→4)-*α*-GalA*p*-(1→4)-*α*-GalA*p*-(1→ connection was speculated to exist through a NOESY correlation between the H5 (δ 3.84) of B and the H1 (δ 4.98) of B. The cross-peak between the H1 (δ 4.98) of B and the C3 (δ 81.58) of F was observed in the HMBC spectrum, and the correlation peak in the NOESY spectrum between the H1 (δ 4.98) of B and H3 (δ 3.6) of F suggesting the presence of a →5)-*α*-Ara*f*-(1→3)-*β*-Gal*p*-(1→ linkage. The signals at δ 4.4/3.68 ppm in the NOESY spectrum and δ104.48/3.68 ppm in the HMBC spectrum were attributed to the correlation between the H1/H3 and C1/H3 of residues F/E, respectively, suggesting the presence of the sugar linkage →3)-*β*-Gal*p*-(1→3,6)-*β*-Gal*p*-(1→. The cross peak at δ 4.52/3.98 ppm in the NOESY spectrum was assigned to the H1/H2 between the residues E/I, indicating the manner of the of →3,6)-*β*-Gal*p*-(1→2,6)-*α*-Man*p*-1→ connection. The correlation signals at δ 5.16/3.96 (NOESY spectrum) and δ 5.16/67.2 ppm (HMBC spectrum) were assigned to H1/H6 and H1/C6 between residues A and I, and the cross peak at δ 5.16/3.96 (NOESY spectrum) and δ 110.45/3.96 ppm (HMBC spectrum) were assigned to H1/H6 and C1/H6 between residues A and E, indicating that the →2,6)-*α*-Man*p*-1→ and →3,6)-*β*-Gal*p*-(1→ residues were terminated by an *α*-Ara*f*-(1→ residue. The manner of the →2,6)-*α*-Man*p*-(1→4)-*β*-Glc*p*-(1→ connection was speculated to exist through an HMBC correlation between the H1 (δ 5.05) of I and the C4 (δ 75.19) of G. The cross-peak between the H1 (δ 4.45) of G and the H4 (δ 3.86) of J in NOESY spectrum revealed the connection of →4)-*β*-Glc*p*-(1→3,4)-*α*-Rha*p*-(1→.

The comprehensive analysis of the monosaccharide composition, methylation analysis, and 1D and 2D-NMR data confirmed that the main backbone of RPP-3a was composed of →4)-*β*-Galp-(1→3,4)-*α*-Rha*p*-(1→[4)-*α*-GalA*p*-(1→4)-*α*-GalA*p*-(1→]_n_ (D-J-[H-H’]_n_). The connection of *α*-Ara*f*-(1→5)-*α*-Ara*f*-(1→3,5)-*α*-Ara*f*-(1→5)-*α*-Ara*f*-(1→5)-*α*-Ara*f*-(1→3)-*β*-Gal*p*-(1→3,6)-*β*-Gal*p*-(1→2,6)-*α*-Man*p*-(1→4)-*β*-Glc*p*-(1→ (A-B-C-B-B-F-E-I-G) was located on the main backbone with the O-4 band of rhamnose and formed the primary branch of RPP-3a. Furthermore, the terminal residue *α*-Ara*f*-(1→ was linked to the primary branch by the O-3, O-6, and O-6 bands of Ara, Gal, and Man, respectively, and formed the secondary branches of RPP-3a. The proposed structure of RPP-3a based on the above analytical results is depicted in Figure 6h.

### 2.7. Antioxidant Activity of RPP-3a

**Figure 7:**
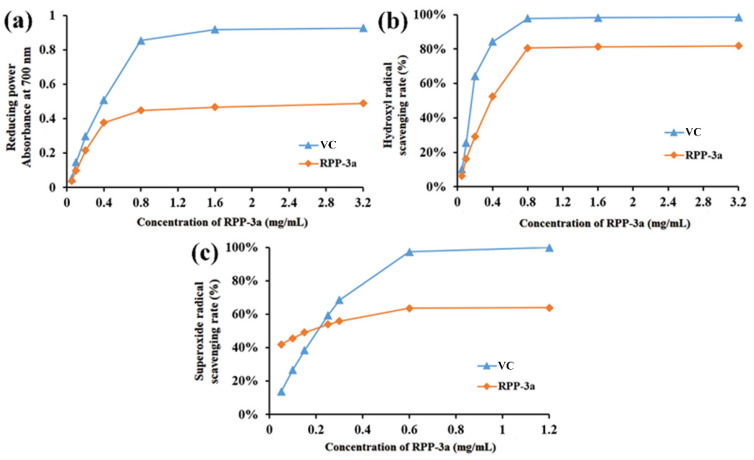
The antioxidant activity of RPP-3a. Reducing power (**a**), hydroxyl (**b**), and superoxide anion radical (**c**) scavenging ability of RPP-3a.

As depicted in Figure 7a, the reducing power of RPP-3a significantly increased with increasing concentrations and reached 0.489 at 3.2 mg/mL, which was approximately half that of vitamin C (VC) at the same concentration. Figure 7b,c depict a comparison between the hydroxyl and superoxide anion radical scavenging ability of RPP-3a with VC. Both the hydroxyl and superoxide anion radical scavenging rates of RPP-3a were concentration-dependent, and the maximal scavenging rate of RPP-3a of hydroxyl and superoxide anion radicals was 81.79% and 63.09%, respectively. However, the superoxide anion radical scavenging rate of RPP-3a was stronger than that of VC at concentrations of 0.05, 0.1, and 0.15 mg/mL. The above results indicate that RPP-3a had a moderate reducing power and strong hydroxyl and superoxide anion radical scavenging ability.

### 2.8. The Effect of RPP-3a on RAW264.7 Macrophage Viability

**Figure 8:**
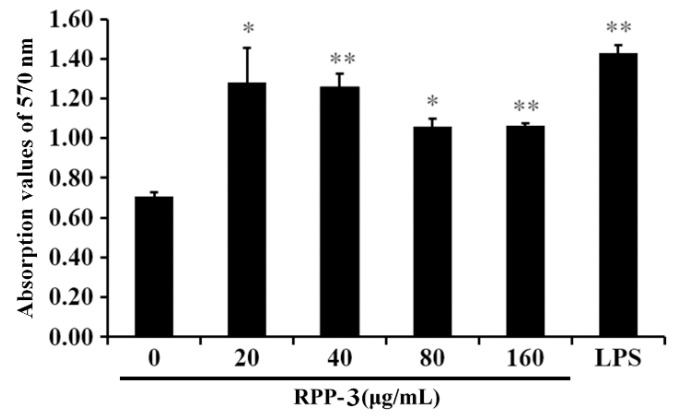
Effect of RPP-3a on the viability of RAW264.7 cells. The viability of RAW264.7 cells was detected with CCK-8 assay kit. Data are presented as the mean ± SD; * *p* < 0.05 and ** *p* < 0.01 vs. the control group.

As shown in Figure 8, the viability of the macrophages in the RPP-3a-treated groups was significantly or extremely significantly different compared to the control group (* *p* < 0.05, ** *p* < 0.01), indicating that RPP-3a significantly promoted the viability of RAW264.7 macrophages in a concentration range of 20–160 μg/mL.

### 2.9. Effect of RPP-3a on Nitric oxide (NO), Tumor Necrosis Factor-α (TNF-α), Interleukin-6 (IL-6), and Interleukin-1β (IL-1β) Production in RAW264.7 Macrophages

**Figure 9:**
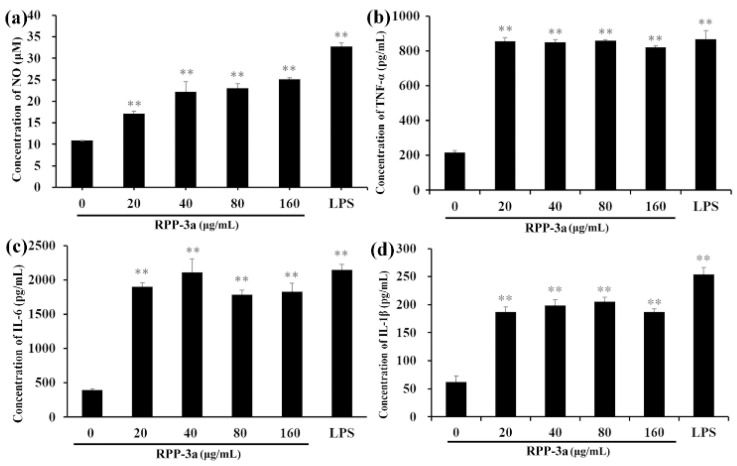
Enhanced effect of RPP-3a on NO and pro-inflammatory cytokine production. NO (**a**), TNF-α (**b**), IL-6 (**c**), and IL-1β (**d**). The concentration of NO was determined using Griess reagent, and the concentrations of TNF-α, IL-6, and IL-1β were determined by ELISA assay kits. Data are presented as the mean ± SD; ** *p* < 0.01 vs. the control group.

As depicted in Figure 9, RPP-3a significantly increased the concentration of NO, TNF-α, IL-6, and IL-1β in the RAW246.7 cells at a concentration range of 20–160 μg/mL (** *p* < 0.01 vs. the control group). The level of NO was significantly increased in a dose-dependent manner (Figure 9a). However, the opposite phenomenon was observed for TNF-α (Figure 9b), IL-6 (Figure 9c), and IL-1β (Figure 9d).

### 2.10. Effects of RPP-3a on the Expression of Inducible Nitric Oxide Synthase (iNOS) and Cytokines in RAW264.7 Macrophages

**Figure 10:**
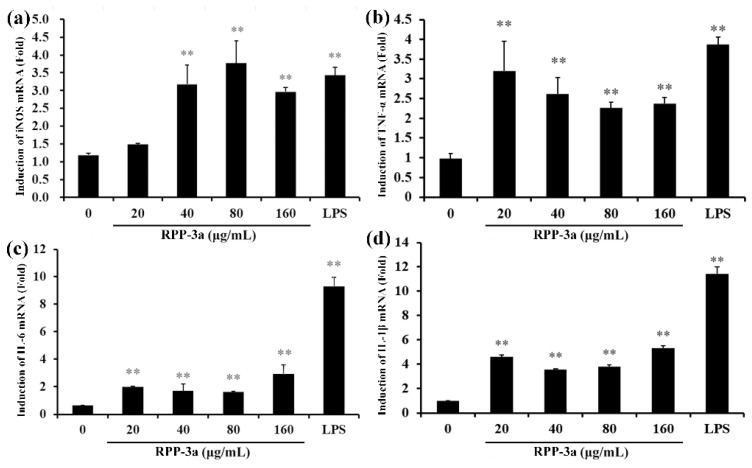
Effect of RPP-3a on mRNA expression of iNOS and cytokines in RAW264.7 macrophages. The mRNA levels of iNOS (**a**), TNF-α (**b**), IL-6 (**c**), and IL-1β (**d**) were measured by qRT-PCR in RPP-3a treated RAW264.7 cells. Data are presented as the mean ± SD, n = 3, ** *p* < 0.01 vs. the control group.

The production of NO is firmly regulated by iNOS [33]. Thus, to further verify the effect of RPP-3a on NO and cytokine secretion in RAW264.7 cells, the mRNA expression of iNOS, IL-1β, TNF-α, and IL-6 was determined by RT-qPCR. As depicted in Figure 10a, RPP-3a promoted the mRNA expression of iNOS in a concentration range of 40–160 μg/mL (** *p* < 0.01 vs. the control group), indicating that RPP-3a enhanced NO production by inducing iNOS expression at the mRNA level. Compared to the control group, the mRNA expression of TNF-α (Figure 10b), IL-6 (Figure 10c), and IL-1β (Figure 10d) was significantly stimulated by RPP-3a (** *p* < 0.01 vs. the control group), suggesting that RPP-3a increased the production of these cytokines in RAW264.7 macrophages at the transcriptional level.

## 3. Discussion

In the present study, an acidic homogeneous polysaccharide RPP-3a, with an Mw of 88,997 Da, was isolated and purified from crude raspberry pulp polysaccharides (Figure 1 and Figure 2). RPP-3a was composed of Rha, Ara, Gal, Glu, Man, and GalA at a molar ratio of 13.1:28.6:16.8:1.4:6.2:33.9 (Figure 4). The results of the structural analysis revealed that the backbone of RPP-3a was composed of repeating units of →4)-*β*-Gal*p*-(1→3,4)-*α*-Rha*p*-(1→[4)-*α*-GalA*p*-(1→4)-*α*-GalA*p*-(1→]_n_ with branches at the C-4 position of Rha. The side chain of RPP-3a, containing two branch levels, was comprised of *α*-Ara*f*-(1→, →5)-*α*-Ara*f*-(1→, →3,5)-*α*-Ara*f*-(1→, →3)-*β*-Gal*p*-(1→, →3,6)-*β*-Gal*p*-(1→, →4)-*β*-Glc*p*-(1→, and →2,6)-*α*-Man*p*-1→ residues (Figure 6 and Table 2). Compared to the previously reported polysaccharides obtained from raspberries, RPP-3a differed in monosaccharide composition, molecular weight, and glycosidic linkages. Ke et al. reported that the polysaccharide separated from *Rubus chingii* Hu, with a molecular weight of 837,820 Da, was composed of Man, Rha, GlcA, GalA, Glc, Gal, Ara, and Fuc, of which GalA and Ara were the major monosaccharides [3]. Another raspberry polysaccharide called RCPI was composed of GalA, Rha, Ara, Xyl, Man, Glc, and Gal at a molar ratio of 1.00:0.15:0.65:0.26:0.11:0.10:0.46. The molecular weight of RCPI was 411,000 Da, and there were seven types of glycosidic linkages in the backbone of RCPI, including →5)-*α*-L-Ara*f*-(1→, →2)-*α*-L-Rha*p*-(1→, →4)-*α*-D-GalA*p*-(1→, →3,6)- *α*-D-Man*p*-1→, →4)-*β*-D-Xyl*p*-(1→, →6)-*β*-D-Glc*p*-(1→, and →3)-*β*-D-Gal*p*-(1→ residues [14]. The variation in the structural features of the polysaccharides derived from raspberries might be due to differences in the geographical environment and climatic conditions, cultivars, extraction and purification procedures, and the different parts of the raspberries used for the extractions [23]. Thus, RPP-3a was inferred to be a novel acid polysaccharide isolated from raspberries.

An increasing number of studies have shown that the biological activity of polysaccharides was closely related to their structural features, including conformation, monosaccharide composition, molecular weight, glycosidic bonds, and degree of branching [28,34]. The high percentage of uronic acid could contribute to the immunomodulatory and antioxidative effects of polysaccharides [35,36]. Ketha reported that the polysaccharides isolated from mung beans were shown to activate macrophages due to the high uronic acid content [37]. Similarly, another in vitro study proved that galacturonic acid played a significant role in the proliferative activity of macrophages [38]. Li reported that the level of uronic acids was positively correlated with the in vitro antioxidant activity of polysaccharides, higher uronic acid content, and higher protective effect against H_2_O_2_-induced oxidative damage in HepG2 cells [7]. Wang et al. also reported that the uronic acid content of polysaccharides exerted significant effects on hydroxyl radical scavenging [39]. Polysaccharides with Rha and Ara exhibited better antioxidant activity than those without these monosaccharides [40]. In this study, GalA, Rha, and Ara were the major monosaccharides of RPP-3a (Figure 4). Thus, RPP-3a might exhibit strong immunomodulatory and antioxidant activities. Besides the monosaccharide composition, the molecular weight might also contribute to the bioactivity of the polysaccharides. Studies have proved that the polysaccharides with lower molecular weight may have significant immunoregulatory effects, as their simpler structural conformation allows them to pass through the cell barrier easier [41]. Interestingly, some high molecular weight polysaccharides also have strong immune regulatory effects due to the presence of more receptors [42]. As for the antioxidative effect, the polysaccharide with a lower molecular weight had better antioxidant activity due to more reductive hydroxyl group terminals [43]. In the present study, the Mw of RPP-3a was 88,997 Da (Figure 2), which was not very large but still might contribute to its immunomodulatory and antioxidant activities. Another factor that affects the biological activity of polysaccharides is the degree of branching. A previous study revealed that the branching degree was closely related to the immunomodulatory activity of pectic polysaccharides, and the immunomodulatory activity could be diminished by removing the branching regions [44]. According to the structural analysis, the side chain of RPP-3a had two levels of branches (Figure 6). Such a complex branching degree with a large amount of reductive hydroxyl group terminals may significantly contribute to its immunostimulatory and antioxidant effects. Overall, it was speculated that RPP-3a might exhibit strong immunomodulatory and antioxidant activity.

Free radicals are by-products produced during metabolic processes in organisms. Excessive free radicals induce various harmful effects in the human body, such as cancer, diabetes, liver injury, skin damage, aging, and fatigue [45]. Hydroxyl radicals, also known as the most reactive free radicals, can react with almost all of the biomacromolecules functioning in living cells [46]. Superoxide anions are a precursor of active free radicals and are relatively weak oxidants. They can generate strong reactive oxidative species and cause oxidant damage and various diseases upon interacting with singlet oxygen or hydroxyl radicals [47]. Fe (III) reduction, reflecting a vital mechanism of polysaccharide antioxidation, could be used as an indicator of electron-donating activity [48]. In the present study, RPP-3a exhibited a moderate reducing power and strong hydroxyl and superoxide anion radical scavenging ability (Figure 7). The presence of large amounts of branched chains and high uronic acid content in RPP-3a may contribute to its antioxidant activity, providing hydrogen or an electron through its hydroxyl groups to free radicals for transformation to a harmless substance and radical chain reaction inhibition [28].

Of the multitude of immune cells, macrophages are the first line of defense with various activities, such as phagocytosis, surveillance, chemotaxis, and the destruction of targeted organisms [26,49]. Research has shown that a macrophage activation is a promising approach to improving host immune capability and strengthening disease resistance [50]. Activated macrophages induced the production of NO and various immunomodulatory cytokines, such as tumor necrosis factors and interleukins, which act as mediators of immune responses to modulate immunity and participate in pro-inflammatory and anti-inflammatory activities [51]. NO, a short-lived gaseous molecule produced by macrophages, is crucial for enhancing the lysis and phagocytosis of macrophages. Thus, it is an excellent biomarker for evaluating macrophage activation [52]. TNF-α is the earliest and a potent pro-inflammatory mediator secreted by activated macrophages, which exerts a variety of biological effects, such as cell proliferation, differentiation, and multiple pro-inflammatory effects [53]. IL-1β acts with TNF-α in inflammatory processes and plays a vital role in the activation of macrophages [54]. IL-6 is a vital bioactive molecule, which can participate in phagocytosis, antigen-presentation, and inflammation regulation, regulating both cellular and humoral immunity [55]. Most polysaccharide-induced immune-enhancing effects are dependent upon the macrophage function [56]. Thus, macrophages and effectors including NO, TNF-α, IL-1β, and IL-6 were utilized to evaluate the immunomodulatory activity of RPP-3a in the present work. The results showed that RPP-3a could significantly promote the viability of macrophages (Figure 8) and increase the production of NO, TNF-α, IL-6, and IL-1β at both the expression and transcriptional levels in RAW246.7 cells (Figure 9 and Figure 10). These results strongly confirmed that RPP-3a possessed significant immune-enhancing activity. However, studies should be undertaken to reveal the underlying immune-promoting mechanisms.

## 4. Materials and Methods

### 4.1. Materials

Frozen raspberry (cultivar name: Autumn Britain) was obtained in December 2020 from Qinghai raspberry agriculture and forestry industrialization Ltd. (Xining, China) and stored at −80 °C until used. RAW264.7 macrophages were supplied by the Cell Bank of the Chinese Academy of Sciences (Shanghai, China). Dulbecco’s Modified Eagle Medium (DMEM), fetal bovine serum (FBS), penicillin/streptomycin (P/S), dextran standards, and lipopolysaccharide (LPS) were purchased from Sigma-Aldrich Co. (St. Louis, MO, USA). The Cell Counting Kit-8 (CCK-8) and NO detection kit were purchased from Beyotime Biotechnology (Shanghai, China). Enzyme-linked immunosorbent assay (ELISA) kits for TNF-α, IL-6, and IL-1β were purchased from the Nanjing Jiancheng Bioengineering Institute (Jiangsu, China). The RNAsimple Total RNA Kit and FastKing cDNA Dispelling RT SuperMix Kit were bought from TIANGEN biochemical technology Co., Ltd. (Beijing, China). The monosaccharide standards, including Rha, Gal, Ara, GalA, Man, Glu, xylose (Xyl), fructose (Fuc), and glucuronic acid (GlcA), were obtained from The National Institute for Control of Pharmaceutical and Biological Products (Beijing, China). DEAE Sepharose Fast Flow gel and Sephadex G-200 gel were purchased from Yangzhou BoRui Saccharide Biotech Co., Ltd. (Yangzhou, China). All chemical reagents were of analytical reagent grade.

### 4.2. Preparation and Purification of Polysaccharides from Raspberry Pulp

#### 4.2.1. Extraction of Crude Polysaccharides

Crude RPPs were prepared according to the method described in our earlier study [11]. In brief, the frozen raspberries were dried, ground, and sieved to remove the seeds. The raspberry pulp powder was defatted with petroleum ether (boiling point, 60 °C) at room temperature for 24 h with continuous stirring and then extracted with 80% ethanol at 60 °C for 2 h to remove the ethanol-soluble constituents. With the assistance of ultrasound, the residue was extracted in distilled water at 60 °C for 2 h. The concentrated portion was deproteinized using Sevage solution. Thereafter, a 4-fold volume of 95% ethanol was added to precipitate the polysaccharides at 4 °C overnight. The crude polysaccharides were collected and freeze-dried for further analyses.

#### 4.2.2. Isolation of RPP-3a

The further purification of RPPs was carried out using DEAE Sepharose fast flow chromatography. The column (7.5 cm × 60 cm) was eluted with deionized water, 0.2 M, and 0.4 M NaCl solution at a flow rate of 15 mL/min, respectively. The eluate was monitored using the phenol-sulfuric acid method [16]. Fractions containing the main elution peak were lyophilized and further purified on a Sephadex G-200 column (2.6 cm × 60 cm) according to the molecular weight, using distilled water as the eluent at a flow rate of 0.5 mL/min. The eluates were monitored and combined using high-performance liquid chromatography (HPLC; RID-10A FRC-10A, Shimadzu, Tokyo, Japan) online detection system equipped with a refractive index detector (RI-502, Shodex, Tokyo, Japan). The refined polysaccharide fractions were concentrated, dialyzed, and freeze-dried for further analyses.

### 4.3. Homogeneity and Molecular Weight Determination

The molecular weight and purity of RPP-3a were determined by the HPGPC method using an LC-10A HPLC system (Shimadzu, Tokyo, Japan) equipped with a BRT105-104-102 series column (8 mm × 300 mm) and a refractive index detector. The experimental settings were as follows: the temperature was maintained at 40 °C, and the mobile phase was 0.05 M NaCl solution with a flow rate of 0.6 mL/min. Dextran of different molecular weights (5000; 11,600; 23,800; 48,600; 80,900; 148,000; 273,000; 409,800, and 670,000 Da) was used as the standards.

### 4.4. Infrared Spectrum Analysis

RPP-3 (2 mg) and 200 mg of dried KBr were ground and pressed into a pellet. Then, the absorbance curves were recorded using an FT-IR spectrophotometer (FT-IR650, Tianjin Gangdong Co., Tianjin, China) in the wavelength range of 4000–400 cm^−1^ [30].

### 4.5. Monosaccharide Composition Analysis

The acetylated derivatives of RPP-3a and monosaccharide standards were converted and analyzed using high-performance anion-exchange chromatography (HPAEC) according to a previously reported method [57,58]. A Dionex ICS-5000 system (Thermo Scientific Co., Waltham, MA, USA) equipped with a CarboPac^TM^PA-20 analytical column (3 mm × 150 mm) and a pulsed amperometric detector was employed in this analysis. The column was eluted with an isocratic NaOH solution (250 mM) for 10 min, followed by NaOAc (500 mM) containing 50 mM of NaOH for another 30 min. The elution temperature, injection volume, and flow rate were set to 30 °C, 5 μL, and 0.3 mL/min, respectively.

### 4.6. Methylation and GC-MS Analysis

RPP-3a was methylated, hydrolyzed, reduced, and acetylated based on the previously reported methods [59]. The acetylated product was dissolved with dichloromethane and examined using a gas chromatography-mass spectrometer (GC-MS; GCMS-QP-2010, Shimadzu, Tokyo, Japan) with an RXI-5 SIL MS chromatographic column (30 m × 0.25 mm × 0.25 μm). The GC temperature program was as follows: the original temperature was 120 °C, which was increased to 250 °C at 3 °C/min and held for 5 min. The detector temperature was 250 °C, and the flow rate of H_2_ was 1 mL/min.

### 4.7. NMR Analysis

RPP-3a (50 mg) was dissolved in 0.5 mL of D_2_O. NMR spectroscopy data were recorded on a 600 MHz spectrometer (Bruker Corp., Fallanden, Switzerland). ^1^H NMR and ^13^C NMR spectra, Distortionless Enhancement by Polarization Transfer 135 (Dept135), ^1^H,^1^H Chemical-Shift Correlation Spectroscopy (^1^H-^1^H COSY), Heteronuclear Multiple Quantum Coherence (HSQC), Nuclear Overhauser Effect Spectroscopy (NOESY), and Heteronuclear Multiple-Bond Correlation (HMBC) experiments were recorded.

### 4.8. Antioxidant Activity

#### 4.8.1. Ferric Reducing Antioxidant Power Assay

The reducing power was tested by the ferric-reducing antioxidant power (FRAP) assay [60]. Approximately 2.5 mL of RPP-3a at different concentrations (0.05, 0.1, 0.2, 0.4, 0.8, 1.6, and 3.2 mg/mL) was mixed with potassium phosphate buffer (2.5 mL, 0.2 M) at pH 6.6 and potassium ferricyanide solution (2.5 mL, 1% (*w*/*v*)). The obtained mixture was kept at 50 °C for 20 min, supplemented with trichloroacetic acid (2.5 mL, 10% (*w*/*v*)), and then centrifuged at 3000 for 10 min. The upper layer was recovered, of which 2.5 mL was mixed with 2.5 mL of distilled water and ferric chloride solution (0.5 mL, 0.1% (*w*/*v*)). After 10 min of incubation, the absorbance was recorded at 700 nm. VC served as the positive control. An equivalent amount of distilled water was used as the control group instead of a polysaccharide solution.

#### 4.8.2. Hydroxyl Radical Scavenging Assay

The scavenging ability of hydroxyl radicals was determined by the previously reported Fenton method [53]. Briefly, 1 mL of polysaccharide solution was mixed with 1 mL of ferrous sulfate solution (9 mM), salicylic acid-ethanol solution (9 mM), and H_2_O_2_ solution (8.8 mM) at various concentrations (0.05, 0.1, 0.2, 0.4, 0.8, 1.6 and 3.2 mg/mL). The mixed solution was incubated in a water bath at 37 °C for 30 min, and then its absorbance was detected at 510 nm. VC served as the positive control. The hydroxyl radical scavenging capacity was calculated using the following equation:

Scavenging rate (%) = 1 − (A_1_ − A_2_)/A_0_ × 100% (1)

where A_0_ is the absorbance of the blank (distilled water instead of the sample), A_1_ is the absorbance of the sample, and A_2_ is the absorbance of the control (distilled water instead of H_2_O_2_).

#### 4.8.3. Superoxide Anion Radical Scavenging Assay

The superoxide anion radical scavenging assay was performed according to a previously reported method [16]. Tris-HCl buffer (2 mL of 50 mM, pH 8.2) was mixed with 1 mL of RPP-3a solution at various concentrations (0.05, 0.1, 0.15, 0.25, 0.3, 0.6, and 1.2 mg/mL). After the addition of 0.2 mL of pyrogallol (6 mM), the mixture was shaken vigorously and reacted at 25 °C for 5 min. Finally, 0.5 mL of 0.1M HCl was added to terminate the reaction. The absorbance of the samples was determined at 320 nm using VC as the positive control. The superoxide anion radical scavenging ability was calculated using the following equation:

Scavenging rate (%) = 1 − A_1_/A_0_ × 100% (2)

where A_0_ is the auto-oxidation rate of the control group, and A_1_ is the auto-oxidation rate of the sample.

### 4.9. Cell Culture

RAW264.7 macrophages were maintained in a DMEM medium supplemented with 10% FBS and 1% P/S in a 5% CO_2_ incubator at 37 °C and humidified atmosphere [61].

### 4.10. Cell Viability

RAW264.7 cells (1 × 10^4^ cells/well) were seeded in a 96-well plate and incubated overnight, and then treated with RPP-3a (20, 40, 80, and 160 μg/mL) or 5 μg/mL of LPS (as the positive control) for 24 h. CCK-8 (10 μL) was added to each well and incubated at 37 °C for another 2 h. The absorbance at 490 nm was measured using a spectrophotometer (Thermo Fisher Scientific Oy, Vantaa, Finland).

### 4.11. Determination of NO, TNF-α, IL-6, and IL-1β Levels

The cell seeding and treatment processes were the same as those described in Section 2.10. NO levels in the cell supernatants were assessed using Griess reagent, and the concentration of TNF-α, IL-6, and IL-1β in the cell culture medium was determined by ELISA assay kits according to the manufacturer’s instructions.

### 4.12. Real-Time Quantitative Polymerase Chain Reaction

RAW264.7 cells (2 × 10^6^ cells/well) were seeded into 6-well plates and incubated overnight at 37 °C. The cells were subsequently exposed to RPP-3a (20, 40, 80, and 160 μg/mL) or LPS (5 μg/mL) for 24 h. The total RNA in the RAW264.7 cells was extracted using the RNAsimple Total RNA Kit (TIANGEN, Beijing, China) and reverse-transcribed into cDNA using the FastKing cDNA Dispelling RT SuperMix Kit (TIANGEN, Beijing, China). The real-time quantitative polymerase chain reaction (RT-qPCR) assay was performed on a LightCycler 96 PCR System (Roche, Basel, Switzerland) with SYBR Green (TIANGEN, Beijing, China). The primer sequences are listed in Table 3. The amplification conditions were as follows: 95 °C of initial denaturation for 15 min, followed by 40 cycles of denaturation at 95 °C for 10 s, annealing at 60 °C for 30 s, and extension at 72 °C for 30 s. The GADPH gene was used as the internal reference gene. The relative expression of the target genes was calculated using the 2^−∆∆Ct^ method.

### 4.13. Statistical Analysis

The data were analyzed by one-way ANOVA, followed by Tukey’s post hoc test using SPSS version 19.0 software (SPSS, Chicago, IL, USA) and expressed as mean ± standard deviation (SD.). A *p*-value of <0.05 (* *p* < 0.05 in the figure) indicated a significant difference, and *p*-value of <0.01 (** *p < 0.01* in the figure) indicated a highly significant difference.

## 5. Conclusions

In this study, a novel homogeneous acidic polysaccharide named RPP-3a was isolated from the raspberry pulp. Its structure was different from another acidic polysaccharide RPP-2a, which was also separated from the pulp of raspberry in our previous study [13]. RPP-3a was composed of rhamnose, arabinose, galactose, glucose, mannose, and galacturonic acid, among which the galacturonic acid content was the highest. The Mw, Mn, and Mp of RPP-3a were 88,997 Da, 59,284 Da, and 70,366 Da, respectively. FT-IR, GC-MS, 1D, and 2D-NMR analyses revealed that the backbone of RPP-3a was composed of repeating units of →4)-*β*-Gal*p*-(1→3,4)-*α*-Rha*p*-(1→[4)-*α*-GalA*p*-(1→4)-*α*-GalA*p*-(1→]_n_ with branches at the C-4 position of rhamnose. The side chain of RPP-3a, containing two levels of branches, was comprised of *α*-Ara*f*-(1→, →5)-*α*-Ara*f*-(1→, →3,5)-*α*-Ara*f*-(1→, →3)-*β*-Gal*p*-(1→, →3,6)-*β*-Gal*p*-(1→, →4)-*β*-Glc*p*-(1→, and →2,6)-*α*-Man*p*-1→ residues. RPP-3a exhibited significant immunostimulatory and antioxidant activities. These results will provide a theoretical basis for the development and utilization of RPP-3a as a potential health-beneficial functional dietary supplement. However, the underlying mechanisms should be investigated in future studies.

## Figures and Tables

**Table 1:** GC-MS analysis of methylated RPP-3a.

Retention Time (min)	Methylated Sugar	Mass Fragments (*m*/*z*)	Linkages Patterns	Molar Ratios
9.523	2,3,5-Me_3_-Ara*f*	43, 71, 87, 101, 117, 129, 145, 161	Ara*f*-(1→	14.73
10.959	2,3,4-Me_3_-Ara*p*	43, 71, 87, 101, 117, 129, 131, 161	Ara*p*-(1→	0.32
13.123	2-Me_1_-Rha*p*	43, 87, 99, 113, 117, 129, 141, 159, 173	→3,4)-Rha*p*-(1→	0.68
14.603	2,3-Me_2_-Ara*f*	43, 71, 87, 99, 101, 117, 129, 161, 189	→5)-Ara*f*-(1→	15.62
16.337	2,3,4,6-Me_4_-Glc*p*	43, 71, 87, 101, 117, 129, 145, 161, 205	Glc*p*-(1→	0.42
17.547	2,3,4,6-Me_4_-Gal*p*	43, 71, 87, 101, 117, 129, 145, 161, 205	Gal*p*-(1→	1.5
18.423	2-Me_1_-Ara*f*	43, 58, 85, 99, 117, 127, 159, 261	→3,5)-Ara*f*-(1→	4.48
19.128	3,4-Me_2_-Man*p*	43, 87, 99, 129, 189	→2,6)-Man*p*-(1→	5.1
21.572	2,3,6-Me_3_-Gal*p*	43, 87, 99, 101, 113, 117, 129, 131, 161, 173, 233	→4)-Gal*p*-(1→	45.41
21.68	2,3,6-Me_3_-Glc*p*	43, 87, 99, 101, 113, 117, 129, 131, 161, 173, 233	→4)-Glc*p*-(1→	1.63
22.414	2,4,6-Me_3_-Gal*p*	43, 87, 99, 101, 117, 129, 161, 173, 233	→3)-Gal*p*-(1→	3.47
24.473	2,3,4-Me_3_-Gal*p*	43, 87, 99, 101, 117, 129, 161, 189, 233	→6)-Gal*p*-(1→	0.57
25.043	2,6-Me_2_-Glc*p*	43, 87, 97, 117, 159, 185	→3,4)-Glc*p*-(1→	0.84
27.92	2,3-Me_2_-Gal*p*	43, 71, 85, 87, 99, 101, 117, 127, 159, 161, 201	→4,6)-Gal*p*-(1→	0.51
29.762	2,4-Me_2_-Gal*p*	43, 87, 117, 129, 159, 189, 233	→3,6)-Gal*p*-(1→	4.72

**Table 2:** Assignment of ^1^H and ^13^C NMR RPP-3a chemical shift values.

Sugar	Linkage Type	H1	H2	H3	H4	H5a/H5	H5b/6a	H6b	OMe	OAc
		C1	C2	C3	C4	C5	C6			
A	*α*-Ara*f*-(1→	5.16	4.13	3.87	4.09	3.76	3.64			
		110.45	82.62	77.97	88.68	62.64				
B	→5)-*α*-Ara*f*-(1→	4.98	4.07	3.96	4.16	3.84	3.75			
		108.83	82.52	78.12	83.6	68.09				
C	→3,5)-*α*-Ara*f*-(1→	5.1	4.24	4.05	4.03	3.84	3.75			
		108.28	80.63	85.39	83.16	67.82				
D	→4)-*β*-Gal*p*-(1→	4.56	3.63	3.72	4.13	3.67	3.74	3.66		
		105.74	73.12	74.64	79.05	75.9	62.11			
E	→3,6)-*β*-Gal*p*-(1→	4.52	3.57	3.68	4.05	3.87	3.96	3.86		
		104.69	71.31	81.5	69.82	74.81	70.76			
F	→3)-*β*-Gal*p*-(1→	4.4	3.47	3.6	3.97	3.64	3.69			
		104.48	71.97	81.58	70.25	76.4	62.26			
G	→4)-*β*-Glc*p*-(1→	4.45	3.24	3.42	3.58	3.54				
		103.63	73.93	76.44	75.19	75.81				
H	→4)-*α*-GalA*p*-(1→(OMe)	4.84	3.66	3.91	4.36	5.05			3.74	1.96
		100.81	69.54	69.79	80.06	72.96	172.19		54.4	22.2
H’	→4)-*α*-GalA*p*-(1→	4.99	3.68	3.92	4.33	4.68				
		100.39	69.8	70.12	79.68	72.92	176.66			
I	→2,6)-*α*-Man*p*-1→	5.05	3.98	3.86	3.6		3.96	3.71		
		99.62	80.12	71.58	68.06		67.2			
J	→3,4)-*α*-Rha*p*-(1→	4.9	3.5	3.97	3.86		1.14			
		99.5	72.58	77.46	78.18		17.8			

**Table 3:** Primers used in RT-qPCR.

Target Gene	Primer
GADPH	forward primer: 5′-GGCCTTCCGTGTTCCTACC-3′
	reverse primer: 5′-TGCCTGCTTCACCACCTTC-3′
TNF-α	forward primer: 5′-GCCAGGAGGGAGAACAGAAACT-3′
	reverse primer: 5′-GGCCAGTGAGTGAAAGGGACA-3′
IL-6	forward primer: 5′-GAGGATACCACTCCCAACAGACC-3′
	reverse primer: 5′-AAGTGCATCATCGTTGTTCATACA-3′
IL-1β	forward primer: 5′-GCCACCTTTTGACAGTGATGAG-3′
	reverse primer: 5′-GACAGCCCAGGTCAAAGGTT-3′
iNOS	forward primer: 5′-CCTGTGAGACCTTTGATG-3′
	reverse primer: 5′-CCTATATTGCTGTGGCTC-3′

## Data Availability

Not applicable.

## Sample Availability

Samples of the compounds are available from the authors.
